# Visual hallucinations in neurological and ophthalmological disease: pathophysiology and management

**DOI:** 10.1136/jnnp-2019-322702

**Published:** 2020-03-25

**Authors:** John O'Brien, John Paul Taylor, Clive Ballard, Roger A Barker, Clare Bradley, Alistair Burns, Daniel Collerton, Sonali Dave, Rob Dudley, Paul Francis, Andrea Gibbons, Kate Harris, Vanessa Lawrence, Iracema Leroi, Ian McKeith, Michel Michaelides, Chaitali Naik, Claire O'Callaghan, Kirsty Olsen, Marco Onofrj, Rebecca Pinto, Gregor Russell, Peter Swann, Alan Thomas, Prabitha Urwyler, Rimona Sharon Weil, Dominic ffytche

**Affiliations:** 1 Department of Psychiatry, University of Cambridge School of Clinical Medicine, Cambridge, Cambridgeshire, UK; 2 Translational and Clinical Research Institute, Newcastle University, Newcastle upon Tyne, UK; 3 University of Exeter Medical School, Medical School Building, St Luke’s Campus, Exeter, UK; 4 Department of Clinical Neurosciences, WT-MRC Cambridge Stem Cell Institute, University of Cambridge School of Clinical Medicine, Cambridge, Cambridgeshire, UK; 5 Health Psychology Research Ltd, Egham, Surrey, UK; 6 Health Psychology Research Unit, Royal Holloway University of London, Egham, Surrey, UK; 7 Faculty of Medical and Human Sciences, The University of Manchester, Manchester, United Kingdom; 8 Institute of Psychiatry, Psychology and Neuroscience, King's College London, London, London, UK; 9 Gateshead Early Intervention in Psychosis Service, Cumbria, Northumberland, Tyne & Wear NHS Foundation Trust, Gateshead, UK; 10 Global Brain Health Institute, Department of Psychiatry, School of Medicine, Trinity College Dublin, Dublin, Ireland; 11 Moorfields Eye Hospital NHS Foundation Trust, London, UK; 12 Institute of Ophthalmology, University College London, London, UK; 13 Brain and Mind Centre and Central Clinical School, Faculty of Medicine and Health, University of Sydney, Sydney, New South Wales, Australia; 14 Clinical Neurologica, Dipartimento di Neuroscienze, Imaging e Scienze Cliniche, Università G.D’Annunzio, Chieti-Pescara, Italy; 15 Bradford District Care NHS Foundation Trust, Lynfield Mount Hospital, Bradford, UK; 16 Gerontechnology and Rehabilitation Group, ARTORG Center for Biomedical Engineering Research, University of Bern, Bern, Switzerland; 17 University Neurorehabilitation Unit, Department of Neurology, University Hospital Inselspital, Bern, Switzerland; 18 Dementia Research Centre, University College London, London, UK

**Keywords:** parkinson's disease, dementia, hallucinations

## Abstract

Visual hallucinations are common in older people and are especially associated with ophthalmological and neurological disorders, including dementia and Parkinson’s disease. Uncertainties remain whether there is a single underlying mechanism for visual hallucinations or they have different disease-dependent causes. However, irrespective of mechanism, visual hallucinations are difficult to treat. The National Institute for Health Research (NIHR) funded a research programme to investigate visual hallucinations in the key and high burden areas of eye disease, dementia and Parkinson’s disease, culminating in a workshop to develop a unified framework for their clinical management. Here we summarise the evidence base, current practice and consensus guidelines that emerged from the workshop.

Irrespective of clinical condition, case ascertainment strategies are required to overcome reporting stigma. Once hallucinations are identified, physical, cognitive and ophthalmological health should be reviewed, with education and self-help techniques provided. Not all hallucinations require intervention but for those that are clinically significant, current evidence supports pharmacological modification of cholinergic, GABAergic, serotonergic or dopaminergic systems, or reduction of cortical excitability. A broad treatment perspective is needed, including carer support. Despite their frequency and clinical significance, there is a paucity of randomised, placebo-controlled clinical trial evidence where the primary outcome is an improvement in visual hallucinations. Key areas for future research include the development of valid and reliable assessment tools for use in mechanistic studies and clinical trials, transdiagnostic studies of shared and distinct mechanisms and when and how to treat visual hallucinations.

## Introduction

Visual hallucinations (VH) and closely-related visual perceptual symptoms (box 1) are common in degenerative diseases of the brain and eye, and their prevalence varies depending on the condition and symptom type. The three predominant clinical contexts in which VH occur as repeated episodes over a prolonged course are the (i) dementias, (ii) Parkinson’s disease (PD), both in its early stages and after progression to PD dementia (PDD) and (iii) eye or visual pathway disease. Prevalence varies across different dementia subtypes with recent estimates of 55% to 78% in dementia with Lewy bodies (DLB), 32% to 63% in PDD, 11% to 17% in Alzheimer’s disease (AD) and 5% to 14% in vascular dementia.^1^ In DLB, well-formed and detailed VH are a core feature and incorporated into diagnostic criteria.^2^ The term Charles Bonnet syndrome is used to describe VH in visual impairment due to eye or visual pathway disease, with prevalence ranging from 15% to 60% depending on the degree of visual loss.^3^ In PD, prevalence of VH is linked to disease duration and dopamine medication, with a more than 80% cumulative prevalence over time.^4^

Box 1 **Glossary of terms**

- Visual hallucination – visual percept not associated with a real object.
- Complex visual hallucination – subtype of visual hallucination whose content is a formed object, face, animal, figure, etc.
- Visual illusion – real object perceived incorrectly. Traditionally used to refer to errors of category identity (eg, pile of cloths seen as a cat).
- Pareidolia – specific subtype of illusion in which faces, objects, etc, are perceived when viewing formless visual stimuli such as clouds, tree-bark, flames or in patterned visual stimuli such as carpets, wallpaper.
- Metamorphopsia – a subtype of illusion used to refer to errors of spatial, temporal perception (eg, seeing a real object distorted, seeing a real object persist in time or at the wrong spatial location).
- Passage hallucination – animal or person passing (en passage), typically brief and in peripheral visual field. Characteristic of Parkinson’s disease psychosis.
- Presence hallucination – sense of someone being close by or beside without an associated visual, auditory or tactile experience. Characteristic of Parkinson’s disease psychosis.
- Minor hallucination – collective term used in Parkinson’s disease to describe illusions, passage hallucinations and presence hallucinations.
- Multimodality hallucination – visual hallucination combined with hallucinations in other senses. Content in different modalities may be perceptually related (eg, figure talking to you) or perceptually unrelated (disembodied voice with content unrelated to figure).
- Pseudohallucination – in neurological literature, a hallucination with insight. In psychiatric literature, a hallucination in the mind’s eye rather than externally projected and related to imagery.
- Full insight – in the context of visual hallucinations, an understanding that the experience is not real. Insight may be absent on the first occasion a hallucination occurs because of its compelling nature but with repeated instances the experience is recognised as false.
- Partial or fluctuating insight – in the context of visual hallucinations, insight is variable and frequently absent at the time the hallucination occurs. Insight may be restored in retrospect.
- Secondary delusion – a false belief related to the visual hallucination (eg, people have been let into the house). Secondary delusions imply impaired insight.

To date, research into the mechanism and treatment of VH has focussed predominantly on these three clinical contexts, with the emergence of parallel, often contradictory, literature. Little consideration has been given to factors common to each condition or how mechanisms might interact when eye disease combines with dementia or PD. The National Institute for Health Research (NIHR) funded a 5 year research programme (SHAPED: Study of visual Hallucinations in Parkinson’s disease, Eye disease and Dementia) to examine VH from a transdiagnostic perspective focussing on these conditions and to develop a unified framework for clinical management based on combined current best practice and treatment evidence. As part of this programme, we undertook an expert-led review process of recent literature and current practice, culminating in a workshop held in April 2018 to formulate consensus guidelines for the clinical management of VH.

### The underlying mechanism of visual hallucinations

The workgroup highlighted two related but distinct aspects of VH mechanism that might inform treatment. One was what brain changes occur at the time of VH (the hallucinating state); the other being what brain changes are associated with a susceptibility to VH (the hallucination trait). Studies of the hallucinating state ideally require the examination of real-time brain changes coincident with VH. Transient activation of the visual association cortex has been found in Charles Bonnet syndrome around the time of onset of VH,^5^ while more widespread changes have been found in PD with de-activation of the visual association cortex and activation of the frontal cortex.^6^ Differences in methodology make it difficult to conclude whether this reflects a difference in the mechanism underlying the VH state in these disorders.

However, most attention has been on brain changes associated with susceptibility to VH. There are three mechanistic models: (i) disturbed balances between top-down and bottom-up aspects of visual perception, (ii) chronic deafferentation causing hyperexcitability to the cortical structures involved in vision and (iii) misattribution of internal imagery.

The first mechanism, the Perception Attentional Dysfunction (PAD) model or related variants,^7–9^ highlights combined impairment in distributed perceptual and attentional networks leading to disturbed balances between top-down and bottom-up processes (or priors and sensory evidence). This has especially been implicated in the aetiology of hallucinations in dementia or PD and proposes that, in combination with poor visual perception, continuous perceptual activity is underconstrained by impaired attentional focus and that the hallucinatory element of a scene is not disconfirmed by discrepant visual input. In contrast, the second, deafferentation-hyperexcitability, model is believed to underlie Charles Bonnet syndrome, and proposes hyperexcitability secondary to chronic functional visual deafferentation, resulting in increased spontaneous activity within the higher visual cortical areas leading to VH.^10^ The third model, derived from psychotic disorders, is similar to PAD in its emphasis on unbalanced generative perception but proposes that hallucinations, whatever their modality, result from a failure to correctly attribute internal events as internal due to failures in source monitoring.^11^

Each model is supported by a range of evidence including: cognitive/higher visual function deficits, functional imaging of task-related activity, resting state metabolism or blood flow, cortical/white matter changes and altered structural and functional connectivity and postmortem neuropathology. The functional and structural changes differ between studies of VH, both within a given condition and across conditions, but may all form part of a distributed network.^12 13^ Pathology involving any part of the network may result in dysfunction that leads to VH, as shown for anatomically distinct lesion sites causing peduncular hallucinations^13^ and VH in PD.^14^

Postmortem evidence has the complication that changes identified may have followed the onset of VH and reflect later disease progression rather than the primary cause of VH. Nevertheless, VH during life in patients with dementia is a strong predictor of Lewy body (LB) pathology at autopsy.^15 16^ In patients with VH associated with PD and DLB, LB pathology is found in the amygdala and parahippocampal gyrus,^17^ superior and lateral frontal cortex (Brodmann area 8/9), inferior/lateral temporal cortex (Brodmann area 20, 21), inferior parietal cortex (Brodmann area 39, 40) and cingulate cortex (Brodmann area 24)(regions pooled from.^18 19^)

Unlike patients with VH in the context of PD with dementia, patients with VH, PD and relative preservation of cognition do not have prominent cortical or hippocampal LB involvement.^20^ VH are also linked to higher amyloid and tau pathology in frontal, parietal and hippocampal areas,^21^ and patients with PD who go on to develop VH have cerebrospinal fluid (CSF) amyloid changes that suggest early AD pathology.^22^ In PD without dementia, the occipital lobe is relatively free of pathology with absent LB and tau pathology and mild amyloid burden irrespective of whether patients experience VH.^23^

### Neurotransmitter systems and VH

In both AD and DLB, there is strong evidence for reduced cholinergic function associated with more frequent VH.^24–27^ This is consistent with evidence from case series in PD and PDD suggesting improvement in VH with cholinesterase inhibitors^28–30^ and improvement in VH, among other neuropsychiatric symptoms, in the secondary analysis of a large-scale clinical trial examining the effect of cholinesterase inhibitors on cognition.^31^

Neurochemical studies of CSF metabolites suggest a negative correlation between the dopamine metabolite homovanillic acid (HVA) and VH in a small number of LBD patients and weak negative correlations with aspartate and taurine in AD.^32^ One suggestion is that VH susceptibility is linked to a specific 3,4-dihydro-xyphenylacetic acid-HVA metabolic deficit, possibly as a result of a common polymorphism in the catechol-O-methyltransferase (COMT) gene. There is also evidence of reduced striatal dopamine transporter binding in patients with PD who go on to develop VH, thought to reflect dysfunctional frontostriatal circuitry and altered inhibitory executive function^33–36^ consistent with the PAD model. This may also help explain why VH in some patients with PD/PDD improve when their dopaminergic load is dropped or partially blocked with drugs such as clozapine or quetiapine.

Postmortem studies also highlight reductions in cholinergic and GABA activity in the absence of major neuronal or synaptic loss, suggesting functional rather than structural changes may contribute to VH.^37^ In PD, increased 5HT2a binding has been linked to VH in postmortem^38^ and in vivo neurotransmitter binding studies.^39^ This may also help explain why the 5HT2a inverse agonist pimavanserin is effective treatment for hallucinations in PD.

In summary, research on the mechanism of VH has largely been confined to studies within a given clinical condition, with a paucity of transdiagnostic research on the wider applicability of mechanisms or interactions between them. It also remains unclear whether mechanisms proposed for complex VH also apply to related perceptual symptoms (eg, illusions, presence hallucinations), or phenomenological variants of complex VH with full, partial/fluctuating and absent insight.

## Visual hallucinations and their management

### Eye disease

Charles Bonnet syndrome VH are associated with diseases affecting the retina, light transmission within the eye (eg, cataract, corneal opacity) or visual pathways and visual cortex. They do not relate to a specific ocular pathology subtype^40^ and can occur in monocular disease. Typical phenomenology includes simple hallucinations (colours and elementary shapes) geometrical patterns, disembodied faces and costumed figures.^41^ Charles Bonnet syndrome risk increases in patients with severe impairment of visual acuity.^3^ The frequency of VH occurrence in Charles Bonnet syndrome reduces over time, but more than 75% of patients will continue to experience hallucinations beyond 5 years after their onset.^42^ Clinical impression, supported by patient surveys^43^ is that Charles Bonnet syndrome is under-recognised with the fear of stigma reducing self-report. Around a third of Charles Bonnet syndrome patients have symptoms requiring clinical intervention beyond reassurance and education (negative outcome Charles Bonnet syndrome).^42^ Compared with patients with eye disease but no VH, Charles Bonnet syndrome adversely affects quality of life.^44^

#### Current practice

For Charles Bonnet syndrome, ophthalmology services will explain symptoms, reassure and signpost for further support and self-help techniques, with limited evidence from a case series that this may reduce VH in some people.^45^ The self-help techniques aim to stop hallucinations at the time they occur and include eye-movements, changing lighting levels to increase visual input and alerting/distraction strategies. If clinically significant through causing distress, referral to other specialities may occur. A staged approach to treatment is used with a health screen and medication review and optimisation of vision (eg, cataract removal).^46^ For people with VH associated with acute visual loss due to macular degeneration, a study of ranibizumab found improvement in 23%, with an association with improved visual acuity.^47^ There is case-report evidence for treatment with anticonvulsants,^48 49^ cholinesterase inhibitors,^50^ 5HT antagonists (ondansetron),^51^ selective serotonin reuptake inhibitors,^52^ atypical neuroleptics,^53^ Yi-Gan San (a Chinese traditional medicine with multiple neurotransmitter effects)^54^ and repetitive transcranial magnetic stimulation.^55^ However, none can be recommended for routine clinical use without further evidence for their efficacy.

### Parkinson’s disease

VH in PD form part of a progressive spectrum of symptoms (PD psychosis) that start with illusions, presence hallucinations and passage hallucinations and progress to formed hallucinations, typically of people and animals.^56^ They are a particular challenge in PD, as treatment for motor symptoms can trigger and worsen VH. They are associated with higher mortality,^57^ which may be linked to antipsychotic use,^58^ and are a stronger predictor of nursing home placement than cognitive or motor symptoms.^59^ The stigma of mental illness may lead to under-reporting.^60^ VH in PD have a significant negative impact on carers, with increasing carer distress as insight into the VH becomes impaired.^60^ Compared with PD patients without VH, patients with VH have reduced quality of life.^61^

#### Current practice

The NICE (National Institute for Health and Care Excellence) 2017 guidelines for PD^62^ recommend a staged approach to treatment, typically undertaken within a PD service. The starting point is a review of medical or pharmacological triggers and a delirium screen with advice on general coping strategies.^63 64^ A reduction in PD medication may be necessary while monitoring for worsening motor symptoms, dopamine withdrawal syndrome or neuroleptic malignant syndrome. Medications should be withdrawn, starting with those most likely to provoke VH, that is, anticholinergics, amantadine and MAO-B inhibitors, followed by dopamine agonists and COMT inhibitors. If VH persist, the cautious withdrawal of levodopa may help.^65 66^ If these strategies are not effective, antipsychotic medications may be considered.^67^ Several randomised controlled trials (RCTs) have shown clozapine to be efficacious, with benefit for VH without worsening motor symptoms.^68 69^ Quetiapine is more widely used than clozapine, but there is less evidence of efficacy.^70–73^ Pimavanserin, a novel antipsychotic with potent inverse agonist activity on the 5HT2A receptor, has emerged as a new potential therapy, with two positive RCTs. Meltzer *et al* 74 reported reduced VH, and Cummings *et al* 75 reported improvements on psychosis scores and caregiver stress. Pimavanserin is licensed as a treatment for PD psychosis in the USA. Rivastigmine and donepezil are used to treat cognitive impairment in PD and may also help reduce VH,^28–30^ although to date there are no RCTs of cholinesterase inhibitors using VH as a primary endpoint.

### Dementia

VH in dementia tend to be of people/children, animals or objects.^76^ Around 50% of patients are significantly distressed by their experiences, with fear and anger being the most common responses.^77^ As core defining features of DLB, they are likely to be present at the point of diagnosis, contrasting with AD where VH occur in later stages of cognitive decline, 5 to 6 years after the onset of dementia.^78^ VH are associated with increased likelihood of nursing home placement.^79^ As in PD, carer impact increases when patient insight becomes impaired.^60^

#### Current practice

VH are managed within dementia services in the wider context of neuropsychiatric symptoms. A staged approach is used with a physical health review, excluding delirium and other medical conditions that can cause VH, and medication review to reduce/stop drugs which may cause or exacerbate VH. Antipsychotics may have benefit^80^ but potential adverse effects of severe antipsychotic sensitivity and mortality mean that they should be used cautiously in LBD. There is some evidence cholinesterase inhibitors reduce neuropsychiatric symptoms, including VH.^26 31 81^ High dose cholinesterase inhibitors have been shown to reduce the frequency of VH in LBD but with increased side effects, needing careful titration under expert supervision.^82^ A study of memantine found reduced hallucinations (which, although not subdivided by hallucination modality, would have mainly been VH) in DLB after 24 weeks treatment.^83^ Transcranial magnetic stimulation and transcranial direct stimulation have been suggested as approaches for VH in LBD, but studies to date have not shown benefit.^84^

### Comorbid disease

Studies of VH in PD or dementia typically exclude patients with eye disease so there is limited data on the prevalence, phenomenology or management of VH in the context of comorbid eye disease. Eye disease may result in an earlier onset of VH in dementia, resulting in the misdiagnosis of AD as DLB.^85^ In PD, eye disease detectable by general ophthalmological examination is not associated with VH;^18 86^ however, more detailed testing with retinal imaging has found reduced retinal nerve fibre layer thickness in PD patients with VH.^86^ Some patients presenting with Charles Bonnet syndrome to ophthalmology clinics may have unrecognised dementia characterised by partial or fluctuating insight into VH.^87^ Case report evidence suggests that optimising vision may help reduce VH in dementia.^88^

## Discussion

The absence of an overarching model for VH in different disorders or evidence-based treatments limited the scope of the recommendations the workgroup could make. The focus of the NIHR programme on PD, dementia and eye disease also meant that VH in other clinical and non-clinical contexts were not covered (schizophrenia/bipolar disorder (s1); bereavement (s2); delirium (s3); sleep-related, medication,^36^ hallucinogen use (s4); peduncular hallucinations (s5); epilepsy (s6 to s7); migraine (s8); visual snow syndrome (s9) - see table 1). However, the consensus view was that where treatment was indicated for these other conditions, similarities in current practice across the core disorders could logically be extended to all conditions. Below we highlight key considerations, the general framework for managing VH and related symptoms, and areas for future research.

**Table 1:** Visual hallucinations in wider clinical and non-clinical context

Condition	Key features
Parkinson’s disease	Occurs throughout PD from early stage disease without cognitive impairment to PDD (see above). Other hallucination modalities can be involved in later stages.
Charles Bonnet syndrome	Eye or visual pathway disease (see above).
Dementia	Includes AD, DLB, PDD, AD, VaD (see above). Other hallucination modalities can be involved.
Comorbid disease	Eye and neurodegenerative disease combined (see above).
Schizophrenia/bipolar disorder	Visual hallucinations are less prevalent than auditory hallucinations in schizophrenia and other psychoses. VH in these conditions rarely occur without auditory hallucinations during the course of the illness and are typically interspersed with unimodal auditory hallucinations.
Bereavement	VH of the deceased can occur as part of normal grief reaction but are less frequent than sensed presence of the deceased.
Delirium	VH are the most common modality of hallucination in delirium where they occur in the context of clouded consciousness, sleep dysregulation and affective symptoms.
Sleep-related	Occasional VH can be normal experiences at the margins of sleep (hypnagogic/hypnopompic hallucinations). They may also present as part of a sleep-disorder (eg, narcolepsy).
Medication side effects	PD medication can precipitate VH but the exact mechanism and its relation to PD neurodegeneration is unclear. Medication with anti-muscarinic effects and opiates are particularly implicated in VH.
Hallucinogen use	Visual perceptual phenomena including visual snow (see below) afterimages, palinopsia and flashback VH may persist after hallucinogen exposure (hallucinogen persisting perception disorder).
Peduncular hallucinations	Complex visual hallucinations caused by brainstem or thalamic lesions. When caused by brainstem lesions, VH are associated with sleep disturbance and eye movement dysfunction. Hallucinations in other modalities can occur.
Occipital/temporal seizures	Ictal phenomenology is based on location of seizure. Simple VH are associated with occipital foci. Complex VH imply involvement of the temporal lobe and limbic cortex.
Migraine	Teichopsia in classical migraine aura and other visual perceptual phenomena.
Visual snow syndrome	A syndrome characterised by persistent dynamic visual noise (snow), palinopsia, entopic phenomena, photophobia and nyctalopia. Associated with migraine.

AD, Alzheimer’s disease; DLB, dementia with Lewy bodies; PD, Parkinson’s disease; PDD, Parkinson’s disease dementia; VaD, vascular dementia; VH, visual hallucinations.

### Case identification

Whatever the underlying condition, help can only be provided for patients with VH if these symptoms have been identified by their clinical team. The workgroup identified the need to address continuing stigma of self-reporting symptoms perceived as indicators of mental illness or dementia. Evidence from eye disease that pre-emptive warning may be effective in reducing distress or emotional impact at VH onset^42^ suggests that low-level information about the possible future occurrence of VH should be provided at the point of eye disease, dementia or PD diagnosis, with signposting to more detailed information which can be accessed at a later stage. Systematic enquiry about the occurrence of VH should be part of routine follow-up to help share responsibility for identifying VH between the patient and care team.

### Threshold for specific treatment intervention

The workgroup noted that VH that are not distressing for the patient or carer do not need treatment beyond general measures, psychoeducation and help in adapting, accepting and living well with symptoms. Typically, this benign VH phase occurs early in the disease, highlighting the importance of keeping VH under review. An important factor defining the threshold at which intervention is required may be the transition from full insight to partial or fluctuating insight, where the patient responds to VH as if they are real at the time they occur, even if insight is restored after the event. This insight-related phenomenological distinction corresponds to that in the neurological literature between pseudohallucinations (defined by intact insight) and hallucinations without insight. The terminology is unsatisfactory as pseudohallucinations carry different implications in the psychiatric literature; however, the conceptual distinction between VH with insight intact in contrast to partial, fluctuating and absent insight states is worth revisiting as it helps mark a transition point for treatment need in all conditions. The workgroup also noted exceptions to the association between insight and treatment need, for example, intervention might be required in the presence of full, continuous insight where VH content is itself distressing or VH become so intrusive they limit function.

### Carers and VH

Another feature of VH common to different conditions is the need to also consider their impact on carers. Factors mediating the increased risk of care home placement with VH are unclear but may include carer distress caused indirectly by VH. The consensus view was that the treatment of VH should extend beyond the patient to provide support and advice for the carer.

### Consensus framework for the management of VH

**Figure 1:**
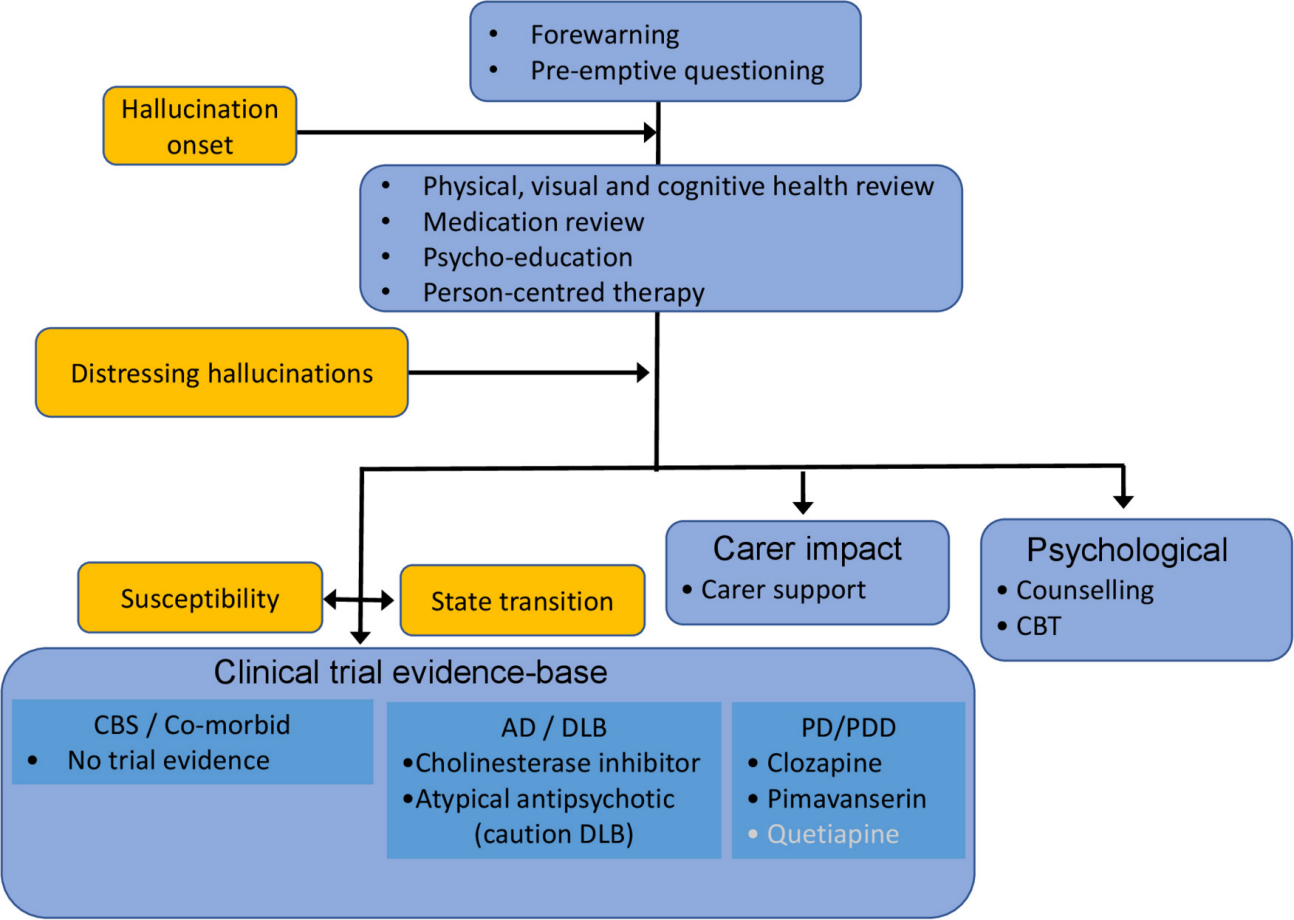
The consensus framework for the management of visual hallucinations in different conditions. Recommendations not supported by meta-analysis are indicated in white. Orange boxes indicate hallucination characteristics and therapeutic targets.AD, Alzheimer’s disease; CBS, Charles Bonnet syndrome; CBT, cognitive behavioural therapy; DLB, dementia with Lewy bodies;PD, Parkinson’s disease; PDD, PD dementia.

The general framework for managing VH and related symptoms is summarised in figure 1. It begins before the onset of hallucinations with forewarning and pre-emptive questioning to encourage their reporting. Once VH are identified, a staged approach is suggested with a review of cognitive and ophthalmological health as well as a physical health/delirium screen. Medication should be reviewed, focussing on anti-muscarinic and opiate drugs and, in PD, dopaminergic therapy. Support including reassurance, psychoeducation, normalisation (explaining VH are part of a disease and have a basis in brain function) and optimisation of visual functioning should be offered. This should be person-centred, identifying the particular triggers and settings that increase the risk of VH and avoiding these situations by planning alternative meaningful and rewarding activities.

VH that become clinically significant by causing distress to the patient or their carers require further intervention. Given the current limitations in both our understanding of the underlying mechanism(s) of VH susceptibility or the neurophysiological changes coincident with VH and the clinical trial evidence base, the workgroup were unable to make definitive medication recommendations. There is a theoretical basis for pharmacological interventions targeting cholinergic, GABAergic, serotonergic or dopaminergic systems and for reducing cortical excitability through non-invasive stimulation or anticonvulsant medication. Treatment might aim to reverse long-term changes associated with VH susceptibility or to reduce the frequency or duration of transient changes coincident with VH.

### Future directions

The working group noted an important methodological challenge for clinical trials or mechanistic studies is the lack of accepted, validated, rating scales for VH or related symptoms. There is a clear need to develop better metrics which extend beyond retrospective collection of questionnaire or scale data to real-world collection of VH as they occur using, for example, new mobile technology or real-time functional data through developments in electroencephalogram telemetry. Measures of VH susceptibility are also required, such as pareidolia tests developed for DLB.^89 90^ Given the importance of insight and its continuity at the decision point for specific intervention, better measures of insight which are sensitive to partial or fluctuating states are required, as well as studies of the cognitive context in which insight becomes impaired, for example, generalised cognitive decline or decline in specific cognitive functions such as self-monitoring or symptom-awareness.^91^

For clinical trials, the workgroup highlighted the lack of standardisation of VH-related outcome measures and the need for trials taking a transdiagnostic, mechanism-based perspective to complement evidence from the traditional condition-specific trials. It remains to be established whether a single treatment approach will be effective in all conditions or whether different treatments will be required with further studies needed to elucidate the underlying mechanism of VH from a transdiagnostic perspective and the role of dysfunctional distributed brain networks. Clinical trials for non-pharmacological approaches are also required, in particular the role of psychological therapies such as rescripting, imagery transformation, desensitisation, cognitive behavioural therapy targeting patient-carer dyads and non-invasive brain stimulation. Both medication and non-pharmacological trials might target longer-term susceptibility or transient changes, either separately or in combination.

## Conclusions

Although the clinical importance of VH and related symptoms has long been recognised, the evidence-base for their pathophysiology or treatment is limited and focusses on single conditions. A wider perspective is required, highlighting key similarities and differences between conditions and taking into account brain changes conferring susceptibility to such symptoms as well as those coincident with their occurrence. In advance of such developments, the workgroup concluded that treatment of VH, irrespective of their clinical context, would benefit from a common management framework and shared priorities for future research.

Additional references are present in online supplementary file 1.

10.1136/jnnp-2019-322702.supp1 Supplementary data
