# Supplementary material for: Visual hallucinations in neurological and ophthalmological disease: pathophysiology and management
Source: J Neurol Neurosurg Psychiatry. 2020 Mar 25;91(5):512–9. doi: 10.1136/jnnp-2019-322702 (PMC7231441; doi:10.1136/jnnp-2019-322702)
Supplement: Supplementary data [file jnnp-2019-322702supp001.pdf]

Online References

s1. Waters, F., et al., *Visual hallucinations in the psychosis spectrum and comparative information from neurodegenerative disorders and eye disease.* Schizophr Bull, 2014. **40 Suppl 4**: p. S233-45.

s2. Rees, W.D., *The hallucinations of widowhood.* British Medical Journal, 1971. **4**: p. 37-41.

s3. Webster, R. and S. Holroyd, *Prevalence of psychotic symptoms in delirium.* Psychosomatics, 2000. **41**(6): p. 519-22.

s4. Halpern, J.H. and H.G. Pope, Jr., *Hallucinogen persisting perception disorder: what do we know after 50 years?* Drug Alcohol Depend, 2003. **69**(2): p. 109-19.

s5. Benke, T., *Peduncular hallucinosis: a syndrome of impaired reality monitoring.* J Neurol, 2006. **253**(12): p. 1561-71.

s6. Panayiotopoulos, C.P., *Elementary visual hallucinations, blindness, and headache in idiopathic occipital epilepsy: differentiation from migraine.* J Neurol Neurosurg Psychiatry, 1999. **66**(4): p. 536-40.

s7. Gloor, P., et al., *The role of the limbic system in experiential phenomena of temporal lobe epilepsy.* Ann Neurol, 1982. **12**(2): p. 129-44.

s8. Klee, A. and R. Willanger, *Disturbances of visual perception in migraine.* Acta Neurologica Scandinavica, 1966. **42**: p. 400-414.

s9. Schankin, C.J., et al., *'Visual snow' - a disorder distinct from persistent migraine aura.* Brain, 2014. **137**(Pt 5): p. 1419-28.
